# Features of chromosomal abnormalities in relation to consanguinity: analysis of 10,556 blastocysts from IVF/ICSI cycles with PGT-A from consanguineous and non-consanguineous couples

**DOI:** 10.1038/s41598-023-36014-6

**Published:** 2023-05-31

**Authors:** Laura Melado, Barbara Lawrenz, Daniela Nogueira, Araz Raberi, Rachana Patel, Asina Bayram, Ibrahim Elkhatib, Human Fatemi

**Affiliations:** 1 Medical Department, ART Fertility Clinics, Marina Village Villa B22 – 23, PO Box 60202, Abu Dhabi, UAE; 2 ART Fertility Clinics, Gurgaon, India

**Keywords:** Clinical genetics, Consanguinity

## Abstract

Consanguineous marriage is defined as marriage between first or second-degree cousins, with high prevalence in many cultures and societies. Descendants from consanguineous unions have an increased risk for genetic diseases. Additionally, in consanguineous couples, chromosomal disjunction during embryogenesis could also be affected, increasing the risk of chromosomal errors. Nowadays, genomic testing allows to identify new genetic syndromes and variants related to copy-number variations (CNV), including whole chromosome, segmental and micro-segmental errors. This is the first study evaluating chromosomal ploidy status on blastocysts formed from consanguineous couples during IVF/ICSI treatments with Preimplantation Genetic Testing for Aneuploidies (PGT-A), compared to non-consanguineous couples. Although consanguine couples were significantly younger, no differences were observed between groups for fertilisation rate, blastulation rate and euploidy rate, once adjusted by age. Nevertheless, the number of blastocysts biopsied on day 5 was lower for consanguine couples. Segmental errors, and aneuploidies of chromosomes 13 and 14 were the most prominent abnormalities in relation to consanguinity, together with errors in chromosome 16 and sex chromosomes when the female partner was younger than 35. Once euploid blastocysts were considered for subsequent frozen embryo transfer, pregnancy outcomes were similar in both groups. The current findings point toward the fact that in consanguine unions, not only the risk of having a child with genetic disorders is increased, but also the risk of specific chromosomal abnormalities seems to be increased. Premarital counselling and tailored reproductive treatments should be offered to these couples.

## Introduction

The definition of consanguinity or inbreeding is an union or marriage between persons who have common biological ancestors, including first and second cousins, double 1st cousins, double 2nd cousins and uncle-niece/aunt-nephew unions^1–3^. Consanguinity has a high prevalence in many cultures and societies worldwide^4^. Twenty per cent of the world population lives in societies where consanguineous marriages are prevalent, and the highest rates have been described in the Middle East (20–50%), reaching > 80% in certain regions^5^. These unions seem to confer social and economic advantages in these societies, such as strengthening family ties, leading to greater marriage stability, and even better support for the female partner^6^.

When the spouses are first degree cousins, they share 1/8th (12.5%) of their genes inherited from a common ancestor, hence their offspring will be homozygous at 1/16th (6.25%) of all loci, meaning they will receive identical gene copies from each parent at these genome sites^7^. These are large runs of homozygosity (ROH) distributed throughout the genome of descendants, representing segments of autozygosity or identical by descent (IBD). As a result, the risk for genetic disorders in consanguineous couples is at least tenfold compared to non-consanguineous couples. The majority are autosomal recessive disorders but, as well, X-linked traits and a considerable number of new genetic syndromes and variants^2^. Furthermore, other chromosomal and microdeletion syndromes, like Prader-Willi syndrome (15q11-q13 deletion), are also related to consanguinity^8^. These genetic disorders have reached epidemic values in the Middle East, with incidences of > 100 cases/100,000 live births per year^9,10^. In addition, consanguinity seems to impair the fertility of subsequent generations^11^, reducing the ovarian reserve of female offspring^12,13^ and increasing the prevalence of severe male factor infertility^13^.

Although genomic testing has evolved and matured in diagnosis of patients with genetic/genomic disorders^14^, there is a lack of information regarding the chromosomal ploidy status of embryos from consanguineous couples, as the attention is attracted to the increased prevalence of genetic disorders in those couples. In the general population, increasing female age has been directly correlated to chromosomal aneuploidy in embryos^15^, leading to implantation failure, miscarriage and the birth of an affected child^16^. The lowest risk for embryonic aneuploidy has been described between ages 26 and 30. Both younger and older age groups had higher rates of aneuploidy and an increased risk for more complex aneuploidies^15^. This information is relevant for understanding of the biology and for better patient counselling.

PGT-A aims to select euploid embryos for subsequent transfer^17^. Ideally, when spouses are carriers of any mutation(s) in a common gene, preconception carrier screening followed by Preimplantation Genetic Testing for Monogenic/single gene disorders (PGT-M) could be implemented for primary prevention of hereditary diseases. However, the presence of aneuploidies involving chromosomes affects embryo implantation potential^18,19^ and up to 50.6% of normal and carrier embryos for monogenic diseases may be aneuploid and not suitable for embryo transfer^20^. Hence, combining PGT-M and PGT-A (Preimplantation Genetic Testing for Aneuploidies) in order to obtain information for both copy number variations (CNV) and monogenic diseases status of an embryo has become the preferable option^21^. However, when only chromosomal disorders are considered, few publications have presented contradictory data on the prevalence of chromosomal abnormalities during pregnancy and after delivery^22,23^.Our aim in this study is to evaluate the prevalence of the chromosomal aneuploidies in the embryos derived from consanguineous couples via PGT-A during their IVF/ICSI treatments. It is important to highlight that, due to its retrospective nature, the herein study includes data derived from PGT for whole and segmental chromosome aneuploidy instead of genomic regions, traditionally used to describe ROH. Additionally, we will look at the effect of consanguinity on fertilisation, embryo development pattern, implantation and miscarriage rates.

## Results

### Descriptive analysis and embryo outcomes

The analysis included 2564 cycles, 2024 (79%) in the non-consanguine group (non-CG) and 540 (21%) in the consanguine group (CG) (Table 1). A total of 10,556 blastocysts with chromosomal information for ploidy were included in the analysis, 8164 (77.34%) from non-consanguineous couples and 2392 (22.66%) from consanguineous couples (Table 2).

**Table 1:** Descriptive analysis for all cycles included.

Cycle characteristics	Total				Non-Consanguineous group			Consanguineous group			t-test
	n	Mean ± SD	Min–Max	95% CI	n	Mean ± SD	95% CI	n	Mean ± SD	95% CI	*p* (t-test)
Age (years)	2564	34.7 ± 6.1	18–50	34.5–35.0	2024	35.1 ± 0.1	34.8–35.4	540	33.3 ± 0.3	32.8–33.8	< 0.001
Partner age (years)	2564	39.4 ± 7.7	21–80	39.1–39.7	2024	39.8 ± 0.2	39.5–40.1	540	38.1 ± 0.3	37.5–38.6	< 0.001
Years of infertility	2531	3.4 ± 3.4	0–25	3.3–3.6	1997	3.3 ± 0.1	3.1–3.4	534	4.0 ± 0.2	3.7–4.3	< 0.001
AMH (ng/mL)	1749	2.5 ± 2.7	0.01–23	2.4–2.7	1303	2.5 ± 0.1	2.4–2.7	446	2.6 ± 0.1	2.3–2.8	0.3964
AFC	2460	11.6 ± 7.9	0–61	11.3–11.9	1945	11.3 ± 0.2	11.0–11.7	515	12.5 ± 0.4	11.8–13.2	0.0015
BMI (kg/m^2^)	1776	28.6 ± 4.8	14.3–45	28.4–28.8	1327	28.5 ± 0.1	28.3–28.8	449	28.7 ± 0.2	28.3–29.2	0.2193
MII inseminated oocytes	2564	10.1 ± 6.5	50	9.9–10.4	2024	9.9 ± 0.1	9.7–10.2	540	10.8 ± 0.3	10.2–11.3	0.005
Fertilized (2PN) oocytes	2564	7.3 ± 5.1	42.0	7.1–7.5	2024	7.2 ± 0.1	7.0–7.4	540	7.8 ± 0.2	7.3–8.2	0.0136
Fertilisation %	2564	73.3 ± 19.3	5.3–100	72.6–74.1	2024	73.4 ± 0.4	72.6–74.2	540	73.0 ± 0.9	71.3–74.7	0.3421
Blastocyst biopsied %	2564	61.1 ± 25.7	4.4–100	60.1–62.1	2024	61.2 ± 0.6	60.0–62.3	540	60.7 ± 1.1	58.6–62.8	0.3522
Euploid %	2564	39.4 ± 35.2	0–100	38.1–40.8	2024	37.7 ± 0.8	36.2–39.3	540	45.8 ± 1.5	43.0–48.7	< 0.001

**Table 2:** Descriptive analysis for included blastocysts distributed by couple consanguinity.

	Non-consanguineous group			Consanguineous group			*p* (t-test)
	n	Mean ± SD	[95% CI]	n	Mean ± SD	[95% CI]	
Patient age at oocyte retrieval (years)	8164	34.8 ± 0.1	34.66–34.92	2392	33.1 ± 0.1	32.86–33.32	< 0.001
Partner age (years)	8164	38.5 ± 0.1	38.35–38.69	2392	36.7 ± 0.1	36.45–36.91	< 0.001
Years of infertility	8092	3.1 ± 0.0	2.98–3.11	2374	3.7 ± 0.1	3.52–3.78	< 0.001
AMH (ng/mL)	5148	3.5 ± 0.1	3.373.55	1900	3.5 ± 0.1	3.33–3-61	0.5392
AFC	7885	15.5 ± 0.1	15.24–15.65	2269	16.7 ± 0.2	16.31–17.07	< 0.001
BMI (kg/m^2^)	5224	28.4 ± 0.1	28.30–28.57	1914	29.1 ± 0.1	28.83–29.28	< 0.001

	n	% ± SD	[95% CI]	n	% ± SD	[95% CI]	*p* (Chi^2^-test)
Day of trophectoderm biopsy							
5	4227	51.8 ± 0.6	50.67–52.85	1188	49.6 ± 1	47.63–51.66	0.192
6	3553	43.4 ± 0.6	42.28–44.43	1083	45.1 ± 1	43.12–47.13	
7	400	4.9 ± 0.2	4.42–5.35	125	5.2 ± 0.5	4.34–6.13	
PGT-A result							
Euploid	3665	44.9 ± 0.6	43.79–45.95	1237	51.6 ± 1	49.61–53.64	< 0.001
Aneuploid	4499	55.1 ± 0.6	54.05–56.21	1155	48.4 ± 1	46.36–50.39	

Consanguineous couples (CG) were significantly younger (33.3 ± 0.3 vs. 35.1 ± 0.1 years; *p* < 0.001) and presented longer periods of infertility (4.0 ± 0.2 vs. 3.3 ± 0.1 years; *p* < 0.001) when compared to non-consanguine couples (non-CG). Antral Follicle Count (AFC) was higher for the CG (12.5 ± 0.4 vs. 11.3 ± 0.2; *p* = 0.001). No differences were found for fertilisation rate (73.0 ± 0.9% vs. 73.4 ± 0.4%; *p* = 0.342) or blastulation rate (60.7 ± 1.1% vs. 61.2 ± 0.6%; *p* = 0.352) in both groups (Table 1).

### Euploidy rate

Crude analysis established the euploidy rate in CG and non-CG, euploidy rate per IVF cycle (Table 1) and euploidy rate per blastocyst biopsied (Table 2). A multivariate analysis was performed to evaluate the euploid rate in CG vs non-CG, adjusted by age. As expected, age had a significant negative impact on euploid rate (Coeff β = −3.05 ± 0.1, *p* < 0.0001, 95%CI: −3.24, −2.85). The consanguinity status of the couple had no significant impact on the euploid rate (45.83 ± 1.46% vs. 37.71 ± 0.79%; Coeff β  = 2.51 ± 1.52, *p* = 0.099, 95%CI: −0.47, 5.45).

### Day of blastocyst biopsy

A total number of 5415 blastocysts were biopsied on day 5, 4636 blastocysts on day 6 and 525 blastocysts on day 7 (Table 2). The percentage of blastocysts that were biopsied on day 5 in the CG (n = 1188, 49.6%) tended to be lower yet not significant compared to the non-CG (n = 4227, 51.8%) (*p* = 0.192). However, the effect of consanguinity status on the percentage of embryos biopsied on day 5 was found to be significant when adjusted for age as a confounding variable: CG had 16% less chance of biopsied blastocyst occurrence on day 5 compared to non-CG (OR 0.84, CI 0.76–0.92; *p* < 0.001).

### Segmental aneuploidies

A total of 974 (17.2%) blastocysts presented segmental aneuploidies (SA), affecting one or multiple (up to 3) chromosomes, irrespective of other whole chromosomal errors. CG presented a significantly higher percentage of segmental aneuploidies compared to non-CG (19% vs. 16.7%, *p* = 0.029) (Table 3).

**Table 3:** Analysis of blastocysts presenting segmental aneuploidies (SA) involving one or more chromosomes. Distribution in the total group and per consanguinity groups.

Aneuploid blastocyst	Total	Consanguinity status, n (%)		*p* Value
		Non-CG	CG	
Aneuploid blastocysts without SA	4680 (82.8%)	3745 (83.2%)	935 (81%)	–
SA—1 segmental error	878 (15.5%)	672 (14.9%)	206 (17.8%)	–
SA—2 segmental error	86 (1.5%)	72 (1.6%)	14 (1.2%)	–
SA—3 or more segmental errors	10 (0.2%)	10 (0.2%)	0 (0%)	–
Total aneuploid blastocysts with SA	974 (17.2%)	754 (16.7%)	220 (19%)	0.029

**Figure 1:**
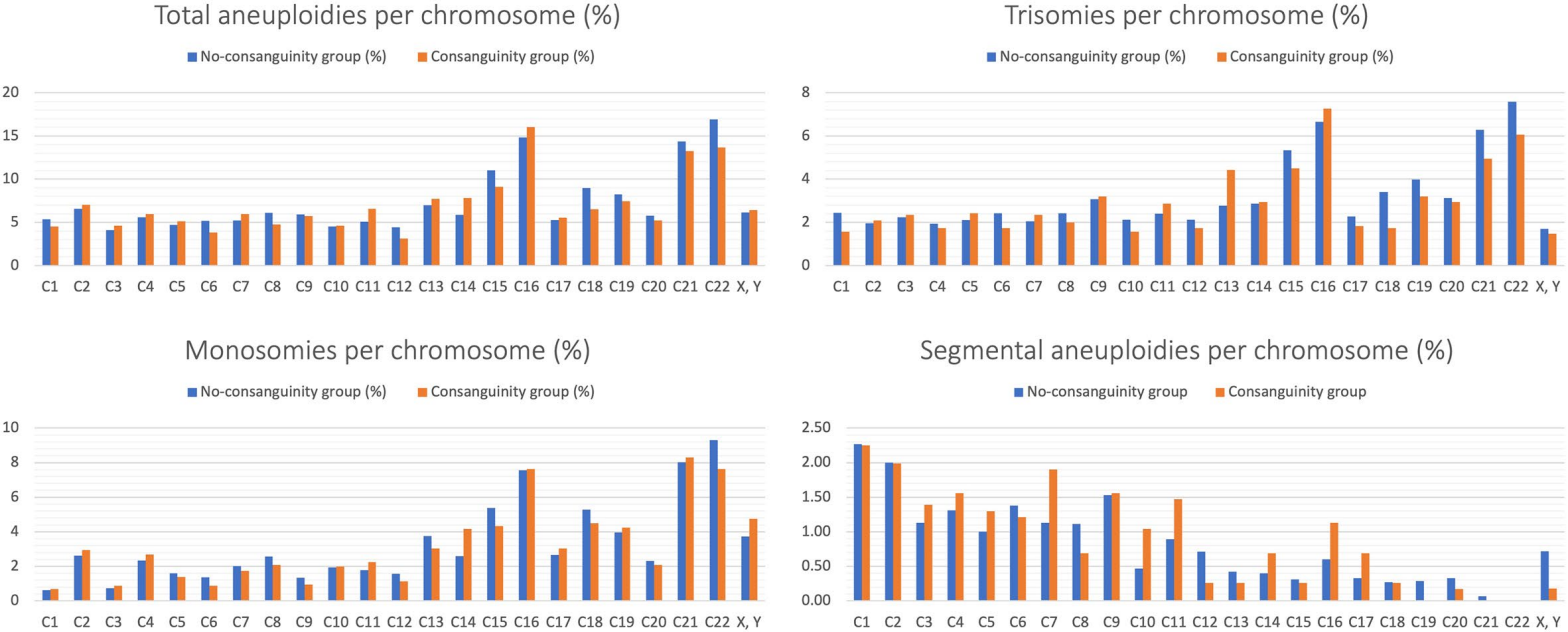
Distribution of the aneuploidies per chromosome.

The chromosomes that were more frequently affected by segmental aneuploidies were chromosomes 1 to 9 in both groups. However, in the CG, chromosomes 1–11 and chromosomes 14, 16 and 17 showed the highest percentage of segmental aneuploidies compared to other chromosomes (Fig. 1) (Suppl Table 1).

### Aneuploidies per chromosome

A significantly higher percentage of chromosomal errors were found in the CG for chromosome 13 (7.71% vs. 6.96%; *p* = 0.019) and chromosome 14 (7.79% vs. 5.85%; *p* = 0.019) compared to the non-CG. However, the percentage of aneuploidies for chromosomes 18 and 22 were higher in the non-CG (8.96% vs. 6.49%, *p* = 0.018; 16.89% vs. 13.68%, *p* = 0.03, respectively) (Suppl Table 1). Figure 1 shows the distribution of the total aneuploidies, monosomies, trisomies and segmental errors per chromosome, considering consanguine and non-consanguine groups.

**Figure 2:**
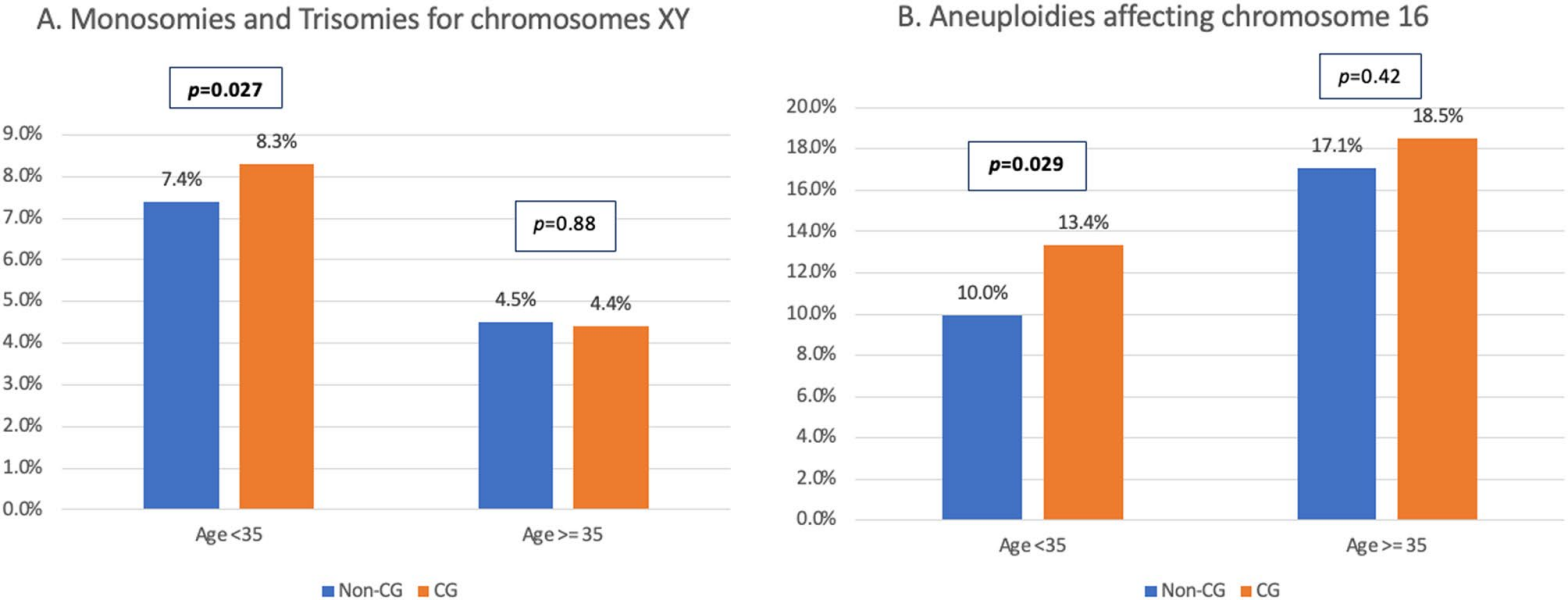
(**A**) Percentage of monosomies plus trisomies affecting chromosomes XY distributed by consanguinity and age groups. (**B**) Percentage of aneuploidies affecting chromosome 16 distributed by consanguinity and age groups.

A further analysis based on age was performed for trisomies involving chromosomes 13, 18 and 21 (Suppl. Table 2), due to the important clinical consequences. Trisomy 13 was significantly more frequent in embryos for the CG when compared to the non-CG (age < 35 years: 59.0% vs. 37.8%; *p* = 0.026; age ≥ 35 years: 56.0% vs. 40.8%; *p* = 0.05). No differences were observed by age categories for trisomies in chromosomes 18 and 21. Regarding aneuploidies for sex chromosomes (X and Y), a higher percentage of monosomies and trisomies accumulatively were observed in the couples from the CG when the age of the female partner was < 35 years (8.3%% vs. 7.4%; *p* = 0.027) when compared to the non-CG < 35 years old. (Fig. 2A).

As chromosome 16 abnormalities are commonly involved in early miscarriages^24^, errors for this chromosome were also evaluated per age categories. For patients < 35 years old, CG revealed a significantly higher percentage of aneuploidies in chromosome 16 compared to non-CG (13.4% vs. 10%; *p* = 0.048). No differences were observed for patients ≥ 35 years old between both groups (CG: 18.5%; non-CG: 17.1%; *p* = 0.88) (Fig. 2B).

### Pregnancy outcomes after euploid frozen embryo transfer

A total of 1660 euploid Frozen Embryo Transfer (eFET) cycles were performed, 364 (21.93%) for the CG and 1296 (78.07%) for the non-CG (Table 4). Regarding patient characteristics, women from the CG were younger (32.5 ± 5.60 vs 33.8 ± 5.46 years, *p* < 0.001) and with higher BMI (27.9 ± 5.16 vs. 26.9 ± 4.80 kg/m^2^; *p* = 0.0018). No differences were observed between groups regarding the number of embryos transferred (CG:1.5 ± 0.5; non-CG:1.4 ± 0.49; *p* = 0.08), day of embryo-biopsy (day 5–6-7; *p* = 0.067), nor endometrial protocol preparation (*p* = 0.099). Pregnancy outcomes were similar between groups (Table 4). However, when euploid Single Embryo Transfers (SET) were evaluated, significantly higher miscarriage rate was observed in the CG (18.8% vs. 12.4%, *p* = 0.048), and Live Birth Rate (LBR) was 5.3% lower in the CG compared to the non-CG, yet not reaching statistical significance (70.7% vs. 76%, *p* = 0.213) (Suppl. Table 3).

**Table 4:** Pregnancy outcomes after Frozen embryo transfers.

Variable	Total	Consanguine group	Non-consanguine group	*p*
N (%)	1660	364 (21.93%)	1296 (78.07%)	
Endometrial preparation				0.099
HRT	985	223 (64.8%)	762 (62.5%)	
NC	564	115 (33.4%)	449 (36.8%)	
Stimulated	14	6 (1.7%)	8 (0.7%)	
Female age, n = 1660 (mean ± SD, range)	33.5 ± 5.43 (19–47)	32.5 ± 5.60	33.8 ± 5.46	< 0.001
Partner age, n = 1659 (mean ± SD, range)	37.1 ± 7.21 (21–80)	35.8 ± 5.65	37.5 ± 7.56	< 0.001
AMH (ng/mL), n = 1417 (mean ± SD, range)	3.3 ± 3.05 (0.01–28.5)	3.3 ± 2.88	3.3 ± 3.0	0.422
BMI (kg/m^2^), n = 1635 (mean ± SD, range)	27.1 ± 4.87 (13.1–43.9)	27.9 ± 5.16	26.9 ± 4.80	0.001
Transfer day, n = 1660				0.670
Day 5 (n, %)	1130 (68.1%)	251 (69.0)	879 (67.8)	
Day 6 (n, %)	509 (30.7%)	110 (30.2)	399 (30.8)	
Day 7 (n, %)	21 (1.2%)	3 (0.8)	18 (1.4)	
Number of embryos transferred (mean ± SD)	1.4 ± 0.49	1.5 ± 0.50	1.4 ± 0.49	0.083
Pregnancy rate, n = 1660 (%)	1169 (70.4%)	261 (71.7%)	908 (70.1%)	0.544
Miscarriage rate, n = 1169 (%)	313 (26.8%)	70 (26.8%)	243 (26.8%)	0.985
Biochemical miscarriage rate, n = 1169 (%)	103 (8.8%)	21 (8.1%)	82 (9%)	0.621
Clinical miscarriage rate, n = 1169 (%)	183 (15.7%)	41 (15.7%)	142 (15.6%)	0.978
Live Birth Rate per pregnancy, n = 1169 (%)	856 (73.2%)	191 (73.2%)	665 (73.2%)	0.985

## Discussion

The offspring from consanguineous couples have large runs of homozygosity (ROH) distributed throughout the genome^25^. The closer the biological relationship between parents, the greater the proportion of the shared alleles and, therefore, the greater the probability that their offspring will receive identical copies of one or more deleterious recessive genes^7,26^. The attention to ROH has arisen with whole genome analysis. The main attention on consanguineous marriage continues to be largely focused on the study of the recessive alleles related to genetic diseases, and recently, it also turned to the relationship between ROH and complex diseases^25,27^. However, there is a lack of information regarding chromosomal errors on embryos before implantation. During the recent years, genomic testing has evolved, allowing to identify new genetic syndromes and variants related to copy-number variations (CNV), including whole chromosome, segmental and micro-segmental errors. The chance that PGT offers to understand the genetic status of the embryos increases the efficiency to exclude the mutations. To the best of our knowledge, this is the first study evaluating chromosomal ploidy status on blastocysts including consanguineous couples using PGT-A during IVF/ICSI treatments and points out the increased risk of chromosomal aneuploidies in consanguineous couples. Segmental errors, aneuploidies of chromosomes 13, 14, and chromosomes 16 and sex chromosomes in patients < 35 years are the frequent abnormalities in relation to consanguinity.

Worse obstetric and perinatal outcomes have been reported in consanguineous couples. Previous publications have shown an increased risk of neonatal and infant death^5^, and the rate among the offspring of consanguineous marriages is approximately 2.5 times higher than among the offspring of unrelated parents^28–30^. In addition, a higher rate of pregnancy complications have been described^31^, including an increased risk of early pregnancy loss^32^. Previously, some authors presented controversial results regarding miscarriage rates among consanguineous populations^2,33^. It is important to notice that early pregnancies and pregnancy losses are easily missed in studies that recruit women later in the first trimester and in populations with little access to healthcare. In addition, preclinical losses are difficult to diagnose and were not considered, resulting in a significant underestimation of prenatal losses^5,32^. An estimated 30% of human conceptions are lost prior to implantation and further 30% post implantation but before the missed menstrual period, that is, in the third or fourth week of gestation. Chromosomal abnormalities are the main factor related to these preclinical and early losses^34^, and implantation failure^16^, yet might be increased as well when parents are related^32^.

Segmental aneuploidies (SA) are generated when a small piece on the p- or q-chromosome arm with any fragment size which is gained or lost during cell division, resulting in sub-chromosomal copy number changes. In the current dataset, 17.2% of the blastocysts analysed presented SA, and CG showed a significantly higher rate (19%) compared to the non-CG (16.7%). SA rates described previously by other groups in general population were similar to the non-CG^35,36^. Babariya et al. described 15.6% of SA on 1327 blastocysts using microarray comparative genomic hybridisation (aCGH)^35^, and Escriba et al.^36^, found an 8.6% of SA out of 3565 blastocysts biopsied using NGS platform. Maternal age is not usually considered a factor related to increased segmental errors in embryos^36^, in line with the herein data, where CG is significantly younger than non-CG (Tables 1 and 2). Instead, segmental errors are believed to result from inability of a cell to complete its cell cycle. In support, the chromosome involved is more likely to be one of the larger chromosomes. Concerning chromosome type, the highest rate of SA in the non-CG was detected in chromosomes 1 to 9, in line with previous studies^35,36^. However, the chromosomes most frequently affected by SA in the CG were chromosomes 1 to 11, chromosomes 14, 16 and 17. It might be possible that the large ROH, seen in the consanguineous offspring, constitute areas of chromosomal instability, with higher risk of generating segmental errors^25,36^. SA are associated to pregnancy losses, accounting for approximately 6% of clinical miscarriages (analysed by FISH)^37^. Also, SA are responsible for complex clinical syndromes and detected in close to 0.05% of new-borns (analysed by FISH and Chromosomal Microarray Analysis)^38^, 5p deletion syndrome (Cri-du-Chat syndrome) and Prader-Willi syndrome (15q11-q13 deletion) with higher rates in offspring from consanguineous couples compared to non-consanguineous parents^8^. However, other chromosomal abnormalities might overlap phenotypes, and the study of sub-chromosomal errors would help to unravel the cause of the syndrome in atypical cases^39^. It is important to note that, in general, segmental aneuploidy appears to be independent of maternal age^36,40^. All of which implies that these segmental errors should be considered when consanguineous couples are counselled.

In addition to the higher rates of segmental aneuploidies, other errors involving the whole chromosome had higher rates in the CG compared to the non-CG. The percentage of aneuploidies for chromosome 13 and 14 were significantly higher, along with errors in chromosome 16 and sex chromosomes for consanguine couples with young maternal age (< 35 years old). Previous authors have suggested the correlation between chromosomal errors and consanguinity, associated to early miscarriages^32^, recurrent miscarriages^41^ and clinical syndromes^8^. As chromosome 16 errors are one of the most common aberrations found in first-trimester miscarriages^42^, the increased incidence of aneuploidies in chromosome 16 in young couples might be a factor involved in the increased risk of miscarriage in consanguine populations. Chromosomes 13 and 14 contain specific genomic regions that may increase their susceptibility to errors during cell division in consanguine couples. Chromosome 13 is relatively small, and chromosome 14 includes repetitive DNA sequences, which can cause problems during DNA replication, recombination, and repair. No need to mention the important clinical implications for trisomy 13 and errors in sex chromosomes, also previously described in inbreed populations^43^. The increased rates of chromosomal errors found in the present dataset implies an important risk for worse obstetric and perinatal outcomes, justifying the indication of PGT-A for this couples, including when female age is below 35 years.

Consanguineous couples tend to marry earlier^5^, and in the present data, they were significantly younger than non-consanguine ones. As expected, age showed an important negative impact in the euploidy rates of the blastocysts analysed. The consanguinity status of the couple had no significant impact on the final euploid rate once adjusted by age, although different chromosomal errors were increased in the aneuploid embryos analysed from the CG. This could be explained due to the higher proportion of blastocysts analysed per couple in the CG compared to the non-CG. Nevertheless, it is worth to highlight that embryo development differed significantly between the studied groups. Despite being younger, a slower blastocyst development was observed in the CG, with a higher number of delayed embryos biopsied on day 6. Consanguine couples had 16% less chance of having a biopsied embryo on day 5 compared to non-CG. Delayed blastocyst formation is a sign of suboptimal embryo development related to lower pregnancy rates, yet not necessarily associated with ploidy status of the embryos^44^. Previous studies have demonstrated that day 6 euploid blastocyst have lower successful outcomes when compared to day 5 euploid blastocyst in frozen embryo transfer^45,46^. However, in the present dataset, when euploid FET were evaluated (Table 4), similar percentage of embryos on day 5 were transferred for the CG and the non-CG, and no difference were seen on the pregnancy outcomes between groups.

Another important factor to be discussed is the significantly higher clinical miscarriage rate seen in the CG when a single eFET was performed (Suppl. Table 3), considering that PGT-A was done for all the blastocyst transferred. Subsequently, live birth rate was approximately 5% less for consanguineous couples and although it did not reach statistical significance, it might be clinically relevant. The fact that more clinical miscarriages were present in CG when SET was performed might also point to undetected small chromosomal abnormalities, which were possibly technically missed. These differences were not observed in the DET sub-group. In addition, other genetic causes or socio-demographic factors, like BMI or endometrial preparation protocol, which may be unequally distributed in the sub-groups, might have an impact on the miscarriage risk once euploid embryo transfers are performed^47^, and should be considered for further investigations.

Despite a large number of cycles and embryos included, the retrospective design is a limitation of the present study. In addition, it includes data derived from PGT for whole and segmental chromosome aneuploidy instead of genomic regions. The hypothesis that the observed differences are related to homozygotization of genomic regions, involving genomic instability during chromosome segregation, cannot be confirmed. The descriptive analysis of the aneuploidies observed should be further evaluated with subsequent prospective studies, including both genomic and cytogenetic analysis, which might help to evidence the genetic tracks of consanguinity in preimplantation embryos. Furthermore, results must be treated with caution before translating into other consanguineous populations, as approximately 85% of the consanguineous couples included are native to the Arabian Peninsula. It is important to mention that, in countries and areas where consanguinity has a high prevalence, it is likely that individuals from these populations share not just a single recent ancestor but also multiple common ancestors (e.g., total genomic homozygosity near or exceeding that seen with first-degree consanguinity, yet the parents have a fairly distant relationship).

The novel description of the chromosomal errors presented in the herein dataset should be taken into consideration altogether with the previously discussed risk for genetic diseases, pregnancy loss and worse obstetric and perinatal outcomes^29,48,49^, in consanguineous couples, which represents an important burden for the families and the healthcare systems. Preimplantation genetic testing (PGT) integrated with in vitro fertilisation (IVF) is a well-established technique, accessible and efficient, offering a reproductive option for consanguineous families to minimise the risk of genetic problems and allowing them to avoid making a decision about termination of an affected pregnancy^50^. The identification of these genetic and chromosomal errors before embryo implantation would redefine the clinical genetic strategy in these populations and would offer opportunities for innovative reproductive health policies tailored to improve the unique needs of consanguineous populations.

In the present study, significantly higher rates of segmental aneuploidies and errors in chromosomes 13 and 14 are observed in consanguineous couples, together with aneuploidies in chromosomes 16 and sex chromosomes when female age was younger than 35. Pre-marital counselling and tailored reproductive treatments should be offer by healthcare providers for consanguineous couples. Preconception carrier screening followed by Preimplantation Genetic Testing for Monogenic/single gene disorders (PGT-M) together with PGT-A could lead directly to primary prevention.

## Methods

### Patients, study design and duration

This is a retrospective observational study, including data from a total of 10,556 blastocysts with chromosomal information for ploidy after preimplantation genetic testing for aneuploidy (PGT-A). Mosaic and non-informative embryos were excluded. Embryos were obtained from 2564 IVF/ICSI cycles of infertile couples, at ART Fertility Clinics UAE, from November 2016 to December 2020. PGT-A indications included advanced maternal age (≥ 35 years old), male factor, recurrent implantation failure (≥ 2), previous miscarriages (≥ 2), poor ovarian reserve as per Bologna criteria^51^, preimplantation genetic testing for monogenic disorders (PGT-M + PGT-A), previous pregnancy diagnosed with chromosomal abnormality, and elective aneuploidy screening.

Trophectoderm biopsy was performed on day 5, 6 or 7 blastocysts for PGT-A using Next Generation Sequencing (NGS) platform for all embryos. Ethical approval was obtained from the Research Ethics Committee (REFA023b) of ART Fertility Clinics Abu Dhabi, UAE. All research was performed in accordance with relevant guidelines/regulations. Informed consent was obtained from all participants.

### Definition of consanguinity

The status of consanguinity (Consanguine group: CG) was defined when couple were first-degree cousins (1st degree consanguinity) or second-degree cousins (2nd degree consanguinity). The status of non-consanguinity (Non-consanguine group: non-CG) was defined as spouses who were not related^1^.

### Ovarian stimulation protocols

Ovarian stimulation was performed by standard GnRH-antagonist-protocols or long-agonist protocols, using recFSH (recombinant Follicle Stimulating Hormone) or HMG (Human Menopausal Gonadotropin) as stimulation medication. The dosage of the stimulation medication was chosen according to the ovarian reserve parameters^52^. From day 5 onwards, the gonadotrophin dose was adjusted according to oestradiol, FSH and progesterone serum levels^53^ and follicular development was assessed by transvaginal ultrasound scan.

Final oocyte maturation was achieved by administration of 5.000–10.000 IU of hCG for long protocols and, in case of antagonist protocols, either 5.000–10.000 IU of hCG, 0.3 mg of GnRH agonist (Triptorelin) or dual trigger (hCG and GnRH-agonist), as per physician’s criteria, as soon as ≥ 3 follicles ≥ 17 mm were present. Oocyte retrieval was carried out 34 or 36 h after.

### Insemination, embryo culture and blastocyst biopsy

Insemination was performed with ICSI or IVF as previously described^54^. All the embryos were incubated in individual 25 μl droplets of Quinn´s Advantage Sequential media (SAGE, MÅlov, Denmark) or single step media (Global) maintained at the same incubation conditions 37 ºC, 5% O2 and 6% CO2. Fertilisation was assessed 17–20 h post-insemination. On day 3 of embryo development (68 h after insemination), media was changed either to extended blastocyst media (SAGE) or refreshed with Global media. Blastocysts were assessed according to Gardner and Schoolcraft^55^. Only full blastocysts with visible inner cell mass and trophectoderm were considered for the blastulation rate. Embryos were cultured until blastocyst biopsy was performed on day 5–7 of preimplantation development.

### Ploidy status of blastocysts by NGS

Biopsy samples were referred to a third partly genetic laboratory (Igenomix, UAE). The PGT-A test was conducted by using the Ion ReproSeq™ PGS Kit (Next Generation Sequencing) for 24 chromosomes aneuploidy screening (Thermo Fisher Scientific, USA). The kit/assay was performed on the Ion Chef™ and Ion S5 System instruments (Thermo Fisher Scientific, Inc, MA, USA). Data analysis was performed with Ion Reporter software, aligning the reads using the human genome build (hg19) (Thermo Fisher Scientific, USA).

All aneuploid blastocysts were further classified as whole-chromosome or sub-chromosome (segmental) aneuploid embryos. Segmental aneuploidies were considered when partial sub-chromosomal gains and losses on the p- or q-chromosome arm with a fragment size > 5 Mb deviated from the standard thresholds for euploidy^36^. This threshold is specifically defined by the manufacturer (see Ion Reporter™ 5.0 Software manual: https://tools.thermofisher.com/content/sfs/manuals/IonReporter_v50_Help.pdf).

### Endometrial preparation (EP) and blastocyst transfer

The EP protocol was chosen according to the physician’s discretion. For a spontaneous ovulatory natural cycle (NC), transvaginal ultrasound scans were performed to monitor follicular growth with serial measurements of serum luteinising hormone (LH), estradiol (E2), and progesterone (P4) levels to accurately determine the ovulation time (automated Elecsys immunoanalyzer, Roche Diagnostics, Mannheim, Germany). Natural micronised progesterone tablets (Endometrin®, Ferring Pharmaceuticals, Switzerland) were commenced on the day of the embryo transfer, every 8 h until pregnancy test^56^.

In hormone replacement therapy (HRT) cycles, patients commenced oral E2 tablets daily from day 3 of menses for 2 days and increased to 6 mg on the fifth day. When an adequate endometrial thickness with a trilaminar appearance was achieved, vaginal progesterone tablets were initiated in the afternoon (day 0) every 8 h and the embryo transfer was programmed 120 h later. Vaginal progesterone was continued until pregnancy test. For the embryo transfer procedure, blastocysts were loaded in a soft pass catheter (GUARDIA™ AccessET Catheter, Cook Medical, USA) in 25 μL of pre-gassed culture medium with the help of a tuberculin syringe and all FET cycles were performed by a physician under abdominal ultrasound guidance. All blastocysts’ FETs were performed 5 days after ovulation was confirmed or on the fifth full day of P4 administration with an average of 120 [115–125] h of P4 exposure between P4 initiation and ET procedure, regardless of the day on which the blastocyst was biopsied.

### Clinical outcomes

A pregnancy was defined 10 days after embryo transfer (ET) by a serum β-hCG value ≥ 15 mIU/mL. Biochemical miscarriage was described by the detection of β-hCG in serum which did not develop into a clinical pregnancy^57^. A clinical miscarriage includes the loss of a clinical pregnancy which takes place between the diagnosis of pregnancy and < 22 weeks’ gestational age. A miscarriage was considered when a spontaneous loss of an intrauterine pregnancy occurred at any gestational age, hence including biochemical miscarriage, clinical miscarriage and any other pregnancy loss that did not end with a live birth. Ectopic pregnancy was only considered in the calculation of pregnancy^58^. Live birth was defined as at least one live birth after 22 weeks. The delivery of a singleton, twin or other multiple birth was registered as one delivery^58,59^.

### Statistical analysis

Patient characteristics were described using mean ± SD, minimum, and maximum values for continuous variables, frequencies and percentages for categorical variables.

Bivariate analysis used a t-test for continuous variable while Chi2 or Fisher exact test was performed for categorical variables to find the differences in consanguine groups. Confidence intervals (95% CI) were presented for each parameter. In addition to each chromosomal abnormalities error, combined errors with monosomy, trisomy and segmental were also analysed. Further, subgroup analysis of chromosomal errors with age (younger vs older) for selected chromosomes. We used two-sided t-test to find the differences in main pregnancy outcomes by consanguinity and single or double FET. The significance level was indicated by *p* < 0.05 in the entire statistical analysis. Data analysis was performed using STATA 17.0, StataCorp LLC.

## Supplementary Information

Supplementary Tables.

## Data Availability

The deidentified participant datasets generated and analysed during the current study are available in the ART Fertility Clinics Institutional data repository, following publication, no end date, on request from Dr Laura Melado Vidales (laura.melado@artfertilityclinics.com).
