# Supplementary material for: Features of chromosomal abnormalities in relation to consanguinity: analysis of 10,556 blastocysts from IVF/ICSI cycles with PGT-A from consanguineous and non-consanguineous couples
Source: Sci Rep. 2023 May 31;13:8857. doi: 10.1038/s41598-023-36014-6 (PMC10232517; doi:10.1038/s41598-023-36014-6)
Supplement: Supplementary file 1 — Supplementary Tables. [file 41598_2023_36014_MOESM1_ESM.docx]

Supplementary Table 1. Distribution of aneuploidies per chromosome and consanguinity groups.

|  | **No-consanguinity group (n=4499)** | **Consanguinity group (n=1155)** |  | **No-consanguinity group (n=4499)** | | | **Consanguinity group (n=1155)** | | |
| --- | --- | --- | --- | --- | --- | --- | --- | --- | --- |
| **Aneuploidies per chromosome** | **n (%)** | **n (%)** | ***p* (Chi2 Test)** | **Monosomy, n (%)** | **Trisomy, n (%)** | **Segmental, n (%)** | **Monosomy, n (%)** | **Trisomy, n (%)** | **Segmental, n (%)** |
| **C1** | 240 (5.33%) | 52 (4.5%) | 0.343 | 28 (0.62%) | 110 (2.44%) | 102 (2.27%) | 8 (0.69%) | 18 (1.56%) | 26 (2.25%) |
| **C2** | 284 (6.58%) | 81 (7.01%) | 0.847 | 118 (2.62%) | 88 (1.96%) | 78 (2.00%) | 34 (2.94%) | 24 (2.08%) | 23 (1.99%) |
| **C3** | 185 (4.1%) | 53 (4.6%) | 0.859 | 33 (0.73%) | 101 (2.24%) | 51 (1.13%) | 10 (0.87%) | 27 (2.34%) | 16 (1.39%) |
| **C4** | 251 (5.57%) | 69 (5.97%) | 0.779 | 105 (2.33%) | 87 (1.93%) | 59 (1.31%) | 31 (2.68%) | 20 (1.73%) | 18 (1.56%) |
| **C5** | 232 (4.71%) | 59 (5.11%) | 0.843 | 72 (1.6%) | 95 (2.1%) | 65 (1.00%) | 16 (1.39%) | 28 (2.42%) | 15 (1.3%) |
| **C6** | 232 (5.16%) | 44 (3.81%) | 0.257 | 61 (1.36%) | 109 (2.42%) | 62 (1.38%) | 10 (0.87%) | 20 (1.73%) | 14 (1.21%) |
| **C7** | 234 (5.19%) | 69 (5.97%) | 0.167 | 91 (2.02%) | 92 (2.04%) | 51 (1.13%) | 20 (1.73%) | 27 (2.34%) | 22 (1.9%) |
| **C8** | 274 (6.09%) | 55 (4.76%) | 0.343 | 115 (2.56%) | 109 (2.42%) | 50 (1.11%) | 24 (2.08%) | 23 (1.99%) | 8 (0.69%) |
| **C9** | 267 (5.93%) | 66 (5.71%) | 0.771 | 60 (1.33%) | 138 (3.07%) | 69 (1.53%) | 11 (0.95%) | 37 (3.2%) | 18 (1.56%) |
| **C10** | 204 (4.53%) | 53 (4.59%) | 0.083 | 87 (1.93%) | 96 (2.13%) | 21 (0.47%) | 23 (1.99%) | 18 (1.56%) | 12 (1.04%) |
| **C11** | 228 (5.07%) | 76 (6.58%) | 0.159 | 80 (1.78%) | 108 (2.4%) | 40 (0.89%) | 26 (2.25%) | 33 (2.86%) | 17 (1.47%) |
| **C12** | 198 (4.4%) | 36 (3.12%) | 0.167 | 70 (1.56%) | 96 (2.13%) | 32 (0.71%) | 13 (1.13%) | 20 (1.73%) | 3 (0.26%) |
| **C13** | 313 (6.96%) | 89 (7.71%) | **0.019** | 169 (3.76%) | 125 (2.78%) | 19 (0.42%) | 35 (3.03%) | 51 (4.42%) | 3 (0.26%) |
| **C14** | 263 (5.85%) | 90 (7.79%) | **0.019** | 116 (2.58%) | 129 (2.87%) | 18 (0.4%) | 48 (4.16%) | 34 (2.94%) | 8 (0.69%) |
| **C15** | 496 (11.02%) | 105 (9.09%) | 0.301 | 242 (5.38%) | 240 (5.33%) | 14 (0.31%) | 50 (4.33%) | 52 (4.5%) | 3 (0.26%) |
| **C16** | 666 (14.81%) | 185 (16.02%) | 0.232 | 340 (7.56%) | 299 (6.65%) | 27 (0.6%) | 88 (7.62%) | 84 (7.27%) | 13 (1.13%) |
| **C17** | 236 (5.25%) | 64 (5.54%) | 0.233 | 119 (2.65%) | 102 (2.27%) | 15 (0.33%) | 35 (3.03%) | 21 (1.82%) | 8 (0.69%) |
| **C18** | 403 (8.96%) | 75 (6.49%) | **0.018** | 238 (5.29%) | 153 (3.4%) | 12 (0.27%) | 52 (4.5%) | 20 (1.73%) | 3 (0.26%) |
| **C19** | 370 (8.23%) | 86 (7.44%) | 0.17 | 178 (3.96%) | 179 (3.98%) | 13 (0.29%) | 49 (4.24%) | 37 (3.2%) | 0 (0%) |
| **C20** | 260 (5.77%) | 60 (5.19%) | 0.766 | 104 (2.31%) | 141 (3.13%) | 15 (0.33%) | 24 (2.08%) | 34 (2.94%) | 2 (0.17%) |
| **C21** | 647 (14.38%) | 153 (13.25%) | 0.283 | 361 (8.02%) | 283 (6.29%) | 3 (0.07%) | 96 (8.31%) | 57 (4.49%) | 0 (0%) |
| **C22** | 453 (16.89%) | 158 (13.68%) | **0.03** | 419 (9.31%) | 34 (7.58%) | 0 (0%) | 88 (7.62%) | 70 (6.06%) | 0 (0%) |
| **X, Y** | 258 (6.15%) | 70 (6.41%) | 0.086 | 157 (3.74%) | 71 (1.69%) | 30 (0.72%) | 52 (4.76%) | 16 (1.47%) | 2 (0.18%) |

Supplementary table 2. Chromosomal errors involving chromosomes 13, 18 and 21.

| \|  \| **Age <35, % (n)** \| ***p*** \| **Age ≥35, % (n)** \| ***p*** \| \| --- \| --- \| --- \| --- \| --- \| \| **Trisomy chromosome 13** \|  \|  \|  \|  \| \| Non-CG \| 37.8% (34) \| **0.026** \| 40.8% (91) \| **0.05** \| \| CG \| 59.0% (23) \| 56.0% (28) \| \| **Trisomy chromosome 21** \|  \|  \|  \|  \| \| Non-CG \| 41.2% (68) \| 0.742 \| 44.6% (215) \| 0.327 \| \| CG \| 36.6% (15) \| 37.5% (42) \| \| **Trisomy chromosome 18** \|  \|  \|  \|  \| \| Non-CG \| 34.0% (32) \| 0.602 \| 39.2% (121) \| 0.149 \| \| CG \| 25.8% (8) \| 27.3% (12) \| |  |  |
| --- | --- | --- | --- | --- | --- | --- | --- | --- | --- | --- | --- | --- | --- | --- | --- | --- | --- | --- | --- | --- | --- | --- | --- | --- | --- | --- | --- | --- | --- | --- | --- | --- | --- | --- | --- | --- | --- | --- | --- | --- | --- | --- | --- | --- | --- | --- |
| **Footnote: Chi2 test (n>5), Fishers Exact test (n<5)** |  |  |

Supplementary table 3. Pregnancy outcomes after euploid frozen embryo transfers, distributed by single (SET) and double embryo transfers (DET).

| **Pregnancy outcomes** | **SET** | | | | **DET** | | | |
| --- | --- | --- | --- | --- | --- | --- | --- | --- |
|  | **n** | **Consanguine group (n=203)** | **Non-Consanguine group (n=769)** | ***p*** | **n** | **Consanguine group (n=161)** | **Non-Consanguine group (n=527)** | ***p*** |
| **Pregnancy rate, n (%)** | 972 | 133 (65.5%) | 499 (64.9%) | 0.868 | 688 | 128 (79.5%) | 409 (77.6%) | 0.611 |
| **Miscarriage rate, n (%)** | 632 | 37 (29.3%) | 115 (24.0%) | 0.252 | 537 | 29 (22.7%) | 118 (28.8%) | 0.17 |
| **Biochemical miscarriage rate, n (%)** | 632 | 10 (7.5%) | 49 (9.8%) | 0.418 | 537 | 11 (8.6%) | 33 (8.1%) | 0.85 |
| **Clinical miscarriage rate, n (%)** | 632 | 25 (18.8%) | 62 (12.4) | **0.048** | 537 | 16 (12.5%) | 80 (19.6%) | 0.059 |
| **Live Birth rate, n (%)** | 632 | 94 (70.7%) | 379 (76.0) | 0.213 | 537 | 97 (75.8%) | 286 (69.9) | 0.201 |
